# Dip‐Pen Nanolithography‐Based Fabrication of Meta‐Chemical Surface for Heavy Metal Detection: Role of Poly‐Methyl Methacrylate in Sensor Sensitivity

**DOI:** 10.1002/smsc.202400459

**Published:** 2024-11-20

**Authors:** Rahma Okbi, Mohammed Alkrenawi, Krishna Kumar Yadav, Dror Shamir, Haya Kornweitz, Yael Peled, Moshe Zohar, Ariela Burg

**Affiliations:** ^1^ Department of Chemical Engineering Sami Shamoon College of Engineering Beer‐Sheva Israel; ^2^ Analytical Chemistry Department NRCN Beer‐Sheva Israel; ^3^ Chemical Sciences Department Ariel University Ariel Israel; ^4^ Department of Electric and Electronic Engineering Sami Shamoon College of Engineering Beer‐Sheva Israel

**Keywords:** dip‐pen nanolithography, heavy metal sensors, meta‐chemical surface, poly‐methyl methacrylate

## Abstract

A meta‐chemical surface is being patterned via dip‐pen nanolithography (DPN) for novel electrochemical heavy metal sensors. The unique feature of DPN allows a precise transfer of desired ink onto various surfaces. Two kinds of sensors are being developed, which differ by the ligand in the poly‐methyl methacrylate (PMMA)‐based ink: 1,8‐diaminonaphthalene (DAN) and D‐penicillamine (D‐PA). The nanosize, the surface‐to‐volume ratio (18.6 and 23.1 μm^−1^ for DAN‐ and D‐PA‐based ink, respectively), and the binding strength between the ligand and the cation (2.21 and −21.37 kcal mol^−1^ for DAN‐ and D‐PA‐based ink, respectively) are found to be the source of their high sensitivity, with limit of detection values of 0.40 and 0.30 ppb for DAN and D‐PA, respectively. According to the DFT calculations, the binding reactions in the presence of PMMA are more exergonic; this indicates that PMMA added to the ink for the patterning process improves the binding between the metals and the ligands. This enhanced binding between the metals and the ligands is a crucial and innovative function of the PMMA that can enhance sensor performance.

## Graphical abstract

Meta‐chemical surface (MCS)‐based 1,8‐diaminonaphthalene and MCS‐based D‐penicillamine are being developed as electrochemical sensors by dip‐pen nanolithography. The surface‐to‐volume ratio and the binding strength between the metals and the ligands affect the sensor sensitivity. The poly‐methyl methacrylate (PMMA) enhances the binding between the metals and the ligands; this is an innovative function of the PMMA that can enhance sensor performance.

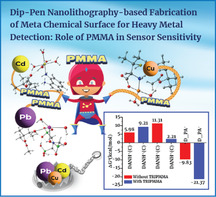

## 1 Introduction

Water is one of the most important resources for all living organisms on earth, especially for human beings;^[^ ^1^ ^]^ however, the scarcity of clean water has always been a challenge.^[^ ^1^ ^]^ At the same time, the rapid growth of the world population and agricultural activities, as well as geological and environmental changes, has introduced pollutants into the water bodies.^[^ ^2^ ^]^ Apart from that, industrialization has released high amounts of metals and other toxic elements into groundwater, increasing their concentrations in drinking water beyond the permissible intake limits suggested by the World Health Organization (WHO).^[^ ^3^ ^]^ It has also been reported that a high intake of metal cations for a long duration can cause death.^[^ ^4^ ^]^ Therefore, early estimation of heavy metals in drinking water before it reaches the public can save lives, and scientists are eagerly working on the development of online heavy metal sensors. The WHO has advised that all metals can be harmful beyond a permissible limit.^[^ ^5^ ^]^ In particular, lead (Pb), cadmium (Cd), and copper (Cu) are more toxic when ingested beyond 0.05, 0.005, and 1.5 ppm, respectively.^[^ ^5^ ^]^ Although Cu shows antimicrobial properties, a large intake can create health issues.^[^ ^6^ ^]^ Interestingly, Pb and Cd often coexist in polluted regions, which challenges their simultaneous detection.^[^ ^7^ ^]^ In any case, it is crucial to know the concentrations of heavy metals in the water.

The present work deals with three heavy metals: Pb(II), Cu(II), and Cd(II). To date, various analytical techniques have been adopted to detect trace amounts of heavy metal and transition‐metal ions; they include flame atomic absorption spectrophotometry,^[^ ^8^ ^]^ electrothermal atomic absorption spectrometry,^[^ ^9^ ^]^ inductively coupled plasma mass spectrometry,^[^ ^10^ ^]^ atomic fluorescence spectrometry,^[^ ^11^ ^]^ and high‐performance liquid chromatography.^[^ ^12^ ^]^ The advantages of these spectroscopic techniques are their low detection limits and their capacity for simultaneous detection of multiatoms. Their disadvantages include the required advanced preparation of the samples, the high levels of expertise needed to execute the techniques, and especially the lack of portability (they cannot be transported for use near a water source). Despite the accuracy of spectroscopic trace detection techniques, its cost and time consumption are the main obstacles to its wide use in households. Therefore, electrochemical methods have emerged as the alternative for portable, cost‐effective, and rapid results with low detection limits.^[^ ^13^ ^]^ Various electrochemical methods such as square‐wave anodic stripping voltammogram (SWASV), chronoamperometry, electrochemical impedance spectroscopy, and differential pulse voltammetry have been adopted for heavy metal sensing.^[^ ^14^ ^]^

It is interesting to note that electrochemical sensors are fabricated for portability and on‐site monitoring; therefore, the size of the electrochemical sensor must be small.^[^ ^15^ ^]^ This causes a reduction in the working electrode's surface, ultimately limiting the sensing performance. Therefore, the working electrode surface area is modified with nanomaterials such as metal oxides,^[^ ^16^ ^]^ carbon‐based materials (carbon nanotubes, graphene, or carbon nitride),^[^ ^17^ ^]^ and metal chalcogenides,^[^ ^18^ ^]^ significantly enhancing heavy metal selectivity and sensitivity. Electrochemical methods show their practical application for detecting multimetals simultaneously by using modified working electrodes.^[^ ^19^ ^]^ Therefore, a new class of working‐electrode modification, such as microfabrication and the lithography technique, can provide an alternative for multiatom detection.^[^ ^20^, ^21^ ^]^ Out of various lithography techniques, dip‐pen nanolithography (DPN) emerges as a maskless direct patterning technique; however, only a few reports are available for electrochemical sensors created by DPN.^[^ ^22^, ^23^ ^]^

In the field of scanning probe lithography, Mirkin et al.^[^ ^24^ ^]^ coined the term “dip‐pen nanolithography (DPN).” This technique gained popularity in electronics due to its direct writing via a “constructive” lithographic tool, which allows hard and soft materials to be patterned down to sizes of less than 50 nm.^[^ ^25^ ^]^ The direct‐writing capability allows DPN to pattern multiple compounds sequentially or in parallel, precisely, and exclusively, whenever and wherever needed. This versatility makes DPN a unique and highly recommended tool for patterning over desired substrates.^[^ ^24^, ^26^, ^27^, ^28^, ^29^, ^30^ ^]^ DPN can execute patterns with a single cantilever holding up to 55 000 pens; this makes the technique very rapid^[^ ^31^ ^]^ and applicable for future integrated semiconductor chip design. DPN was previously used in electronics, biotechnology, optical, and materials science.^[^ ^32^, ^33^ ^]^ One of its prime achievements is the creation of patterns with high resolution and accuracy. Another is the creation of nanoclusters with a high surface‐to‐volume ratio (S/V),^[^ ^22^, ^23^, ^34^ ^]^ which will favor their performance in sensing heavy metals. For the first time, alkanethiol molecules were patterned over a gold substrate with a 15 nm resolution using the DPN method.^[^ ^35^ ^]^ Although DPN has many advantages, to our knowledge, we have been the first group to start using DPN‐modified electrodes for heavy metal electrochemical sensors. This was achieved by developing a meta‐chemical surface (MCS) as a working electrode.^[^ ^22^ ^]^ Earlier, we utilized ink based on nitrilotris(methylene) tri‐phosphonic acid (NTPH), in the presence of acetonitrile (ACN) and poly‐methyl methacrylate (PMMA), for the modification of a platinum‐based surface.^[^ ^22^ ^]^ It is interesting to note that PMMA is a polymer broadly utilized in patterning due to its remarkable characteristics such as high transparency, weather resistance, biocompatibility, and cost‐effectiveness.^[^ ^36^ ^]^ PMMA is known for its use in optical components and optoelectronics devices, mainly due to its exceptional optical transparency in a broad range of wavelengths from near‐UV to near‐infrared.^[^ ^36^ ^]^ PMMA is also a crucial thermoplastic material.^[^ ^37^ ^]^ Its versatility makes it an ideal material for various technological and productive applications.^[^ ^36^, ^37^ ^]^

It has been found that the (S/V) ratio or size of the patterned clusters has a considerable impact on the oxidation peak current of Pb(II) sensing. A higher oxidation peak current occurs due to a larger exposed area (high S/V) for metal deposition during the preconcentration, which can be stripped during SWASV. In the present work, we fabricated an MCS over an Au surface, and the patterned MCS electrodes showed remarkable chemical properties that exist in the material's bulk form but are completely altered when patterned.^[^ ^34^ ^]^ Due to interference, the simultaneous electrochemical detection of heavy metals in multication solutions is challenging. The redox peaks can overlap, especially if the cations have a similar nature, and sometimes cations also form intermediate alloys, which could also present redox peaks.^[^ ^38^ ^]^

In the present work, we used two different ligands, 1,8‐diaminonaphthalene (DAN) and D‐penicillamine (D‐PA), as the active species of the sensor. Both ligands have more than one functional group that can bind heavy metals (log K_1_ of D‐PA equal to 13.0, 11.4, and 16.5 for Pb(II), Cd(II), and Cu(II), respectively).^[^ ^39^ ^]^ Therefore, each ligand can bind simultaneously to the multications and the conductive surface (D‐PA can bind the Au‐electrode surface with the sulfide group).^[^ ^40^ ^]^ It is interesting to note that poly1,8‐diaminonaphthalene (poly(DAN)) has been previously used for the extraction of several heavy metals, such as Cu(II), Hg(II), Pb(II), and Cd(II), by forming their complexes.^[^ ^41^, ^42^ ^]^ At the same time, the conductivity of poly(DAN) makes it an electrochemical catalyst for metal trapping.^[^ ^42^ ^]^ Several works have reported metals binding to poly(DAN); however, none have calculated the stability constant for poly(DAN) or DAN. In one report, *K*_d_ values were reported, indicating that heavy metals such as cadmium and lead can bind to poly(DAN).^[^ ^43^ ^]^

Therefore, in this current study, we focused on detecting cations using fabricated MCSs with a surface of DAN and D‐PA ink over a gold substrate. SWASV was performed for the detection of Pb(II) in the presence of Cd(II) using DAN‐based ink, and Pb(II) in the presence of Cu(II), using D‐PA‐based ink.

## 2 Results and Discussion

**Figure 1:**
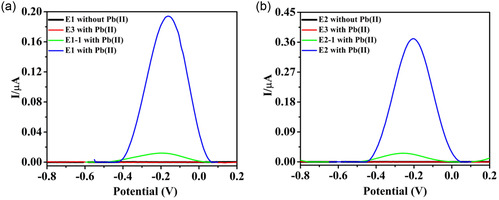
SWASV of control experiments using a) E1 electrode (DAN‐based ink) and b) E2 electrode (D‐PA‐based ink). The solution included 0.50 ppb Pb(NO_3_)_2_, 100 μM ionic strength at pH 3.0. Accumulation for 60 s at −1.1 V and SWASV detection conditions: step potential = 5 mV; amplitude = 100 mV; and frequency = 25 Hz.

**Figure 2:**
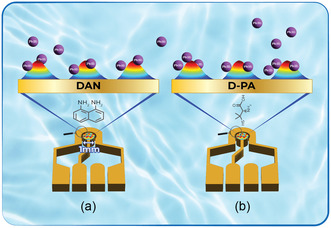
Binding process description between the lead cations and the nanoclusters. a) Patterned nanoclusters with DAN‐based ink on E1 electrode. b) Patterned nanoclusters with D‐PA‐based ink on the E2 electrode.

The electrochemical SWASV method was used to study MCS efficiency and sensitivity. Before the sensing performance, the effect of the solution's pH was studied. It was found that HNO_3_‐based supporting electrolytes at pH 3 showed a higher oxidation current than the acetate buffer at pH 4.5 (Figure S1, Supporting Information). Therefore, all subsequent experiments were carried out using HNO_3_ at pH 3. Once the pH had been defined, control experiments were performed with electrodes E1, E1‐1, E2, E2‐1, and E3, where the solution included Pb(II) (**Figure** **1** and **Table** **1**). It was found that no oxidation peak appeared with the E3 electrode (PMMA‐patterned electrode without a ligand) for Pb(II), and no peak appeared for E1 and E2 without any heavy metals (Figure 1). In the present work, three kinds of solutions were detected: Pb(II) solutions, solutions that included Pb(II) and Cd(II), and solutions that included Pb(II) and Cu(II). The first step in the electrochemistry process is a deposition process where heavy metals in the solutions are reduced following reaction 1. The second stage in the SWASV is the oxidation, reaction 2. After the oxidation process, cations could bind to the nanoclusters (reaction 3, **Figure** **2**) and the Au surface. Each nanocluster was made from a ligand, PMMA, and ACN. However, no peaks are observed if they bind to the Au surface (E‐3, Figure 1).

(1) $$M{\left(\text{II}\right)}_{\text{aq}}+2e^{-}\to M_{\text{aq}}^{0}\;;M=\text{Pb, Cd, Cu}$$

(2) $$M_{\text{aq}}^{0}\to M{\left(\text{II}\right)}_{\text{aq}}+2e^{-}$$

(3) $$M{\left(\text{II}\right)}_{\text{aq}}+\text{ligand }\to M\left(\text{II}\right)\text{ligand};\;\text{ligand}=D_{-}\text{PA},\text{DAN}$$

**Table 1:** Name of electrodes, ink, and statistical data obtained from MATLAB analysis patterned with 6.6 μm pitch.

Electrodea)	Inkb)	Cluster height [nm]	Base diameter [μm]		Elemental volume [μm^3^]	Surface area‐to‐volume ratio (S/V) [μm^−1^]
			Minor axis	Major axis		
E1	Ink1	302	1.77	3.13	0.30	18.6
E1‐1	Ink1	Coated by DPN				
E2	Ink2	96.8	2.92	5.43	0.57	23.1
E2‐1	Ink2	Coated by DPN				
E3	Ink3	208	1.70	2.30	0.17	17.0

a) The patterning and the patterning coated processes are described in the experimental section.

b) The ink composition table is detailed in Table 4.

Small oxidation peaks were observed using coated electrodes E1‐1 and E2‐1 in solutions that included Pb(II), compared to the current that appeared using E1 and E2 electrodes (Figure 1a,b). These results confirmed that the patterned electrodes exhibited better sensitivity to the Pb(II) ions. This is due to the higher amount of exposed electrocatalytic area, resulting in higher S/V of the nanoclusters compared to coated electrodes.^[^ ^22^, ^23^, ^34^ ^]^

**Figure 3:**
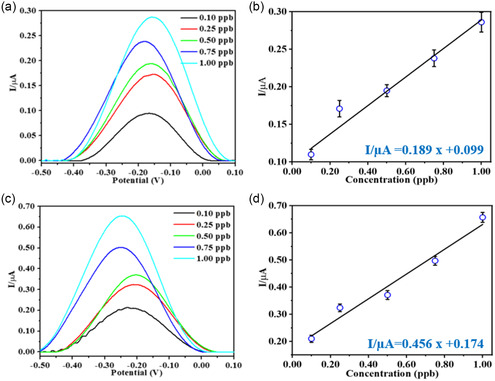
SWASV of different concentrations of Pb(II) a) SWASV of E1, b) calibration points for E1, c) SWASV of E2, and d) calibration points for E2 (100 μM ionic strength, pH: 3.0, E deposition: −1.1 V, deposition time: 60 s, step potential: 5 mV, Amplitude: 100 mV, Frequency deposition: 25 Hz).

**Figure 4:**
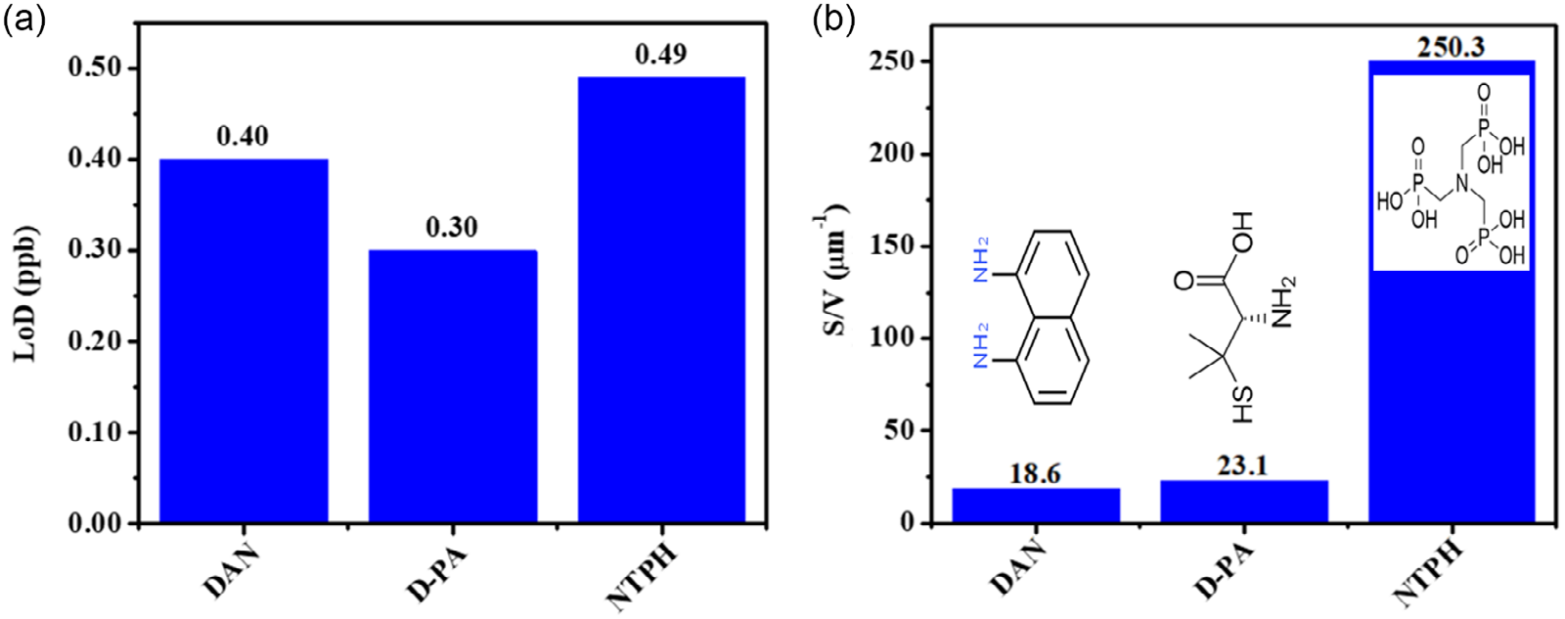
a) LoD's of Pb(II) on electrodes with different patterned ligands. b) Averaged S/V ratio of the nanoclusters on the patterned electrodes.

E1 and E2 electrodes are patterned with different types of ligands; therefore, the oxidation potential of lead is different for each electrode (−0.17 and −0.20 V for E1 and E2, respectively). The SWASV results for E1 and E2 electrodes showed that the oxidation peak current increased as the Pb(II) concentration increased (**Figure** **3**a,c). The oxidation peak current was plotted against the Pb(II) concentration (Figure 3b,d). The obtained data points are fitted with *I*/μA = 0.189 *x* + 0.099; (*R* ^2^ = 0.967) and *I*/μA = 0.456 *x* + 0.174; (*R* ^2^ = 0.969) for E1 and E2, respectively. The obtained equations were used for the limit of detection (LoD) calculations for Pb(II), using the standard LoD equation, which is defined as LoD = 3*s* m^−1^ (*s = *standard deviation in the intercept, *m* = slope).^[^ ^13^ ^]^ The obtained LoDs were 0.40 and 0.30 ppb for E1 and E2, respectively (**Figure** **4**a), and showed lower values than earlier reports, where the electrode surface was modified with carbon, and ≈2 ppb LoD was obtained for Pb with ≈5 min preconcentration time.^[^ ^44^ ^]^ Various microscale sensors fabricated using copper^[^ ^45^ ^]^ and gallium oxide^[^ ^3^ ^]^ showed LoDs of ≈4 and ≈17.1 ppb, respectively. In our earlier work, we showed that the surface of electrodes can be modified by using DPN to pattern NTPH over a platinum surface, and we obtained 0.49 ppb LoD,^[^ ^22^ ^]^ which is ≈1.22 and ≈1.63 times higher than E1 and E2, respectively.

**Figure 5:**
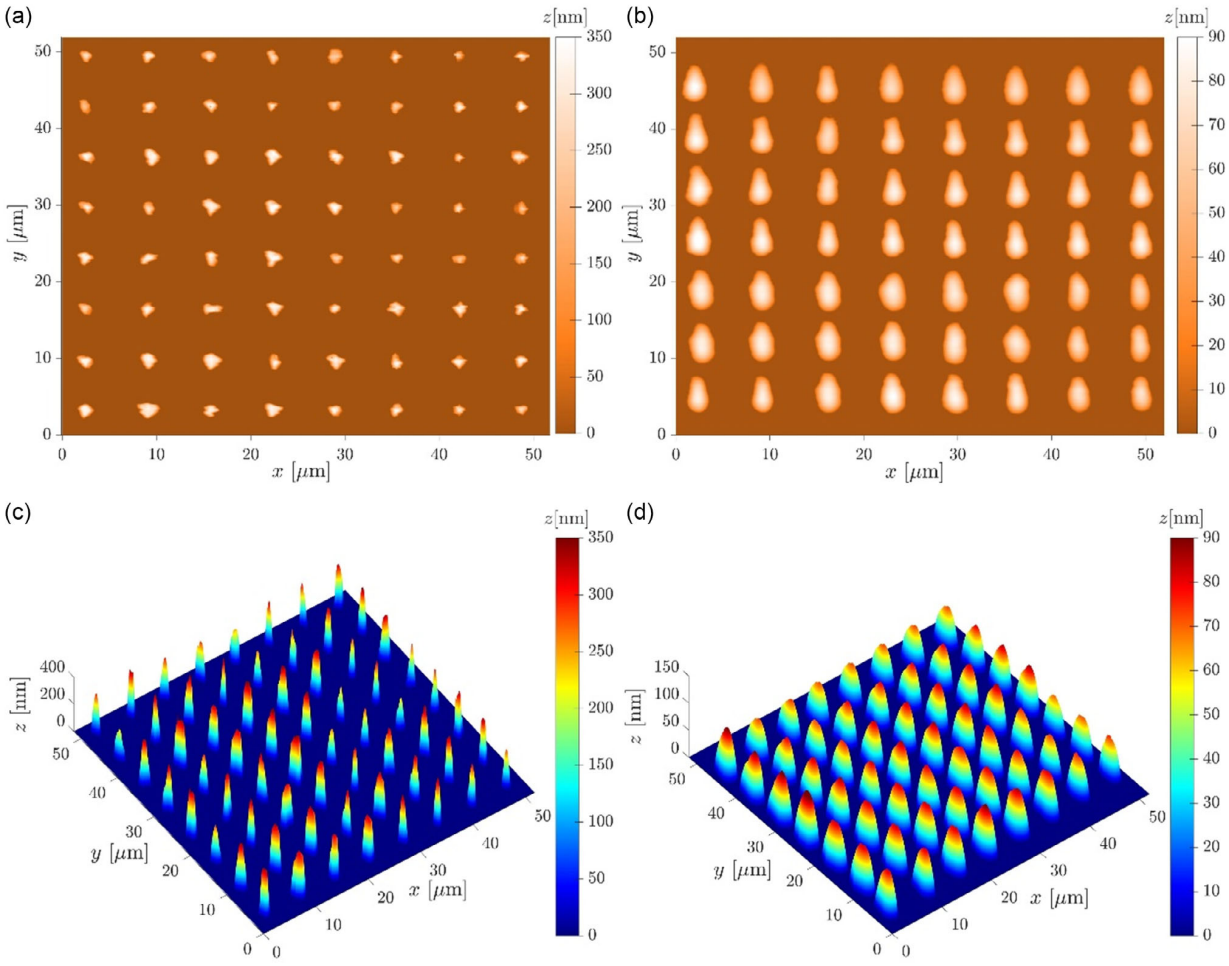
AFM images of a) E1 and b) E2; MATLAB processed AFM images of c) E1 and d) E2.

In order to explain the difference between the LoD values, each MCS was scanned by atomic force microscope (AFM) (**Figure** **5**a,b) and the effect of the nanocluster's S/V ratio on the sensor sensitivity was studied. The S/V ratio of the nanoclusters was extracted from the AFM data of E1 and E2 (Figure 4b and 5a,b). Here, with a dedicated MATLAB code, we utilized AFM data to calculate various parameters such as the nanocluster's height, S/V ratio, cluster base, and elemental volume (Table 1). It is observed that the S/V ratio equals 18.6 and 23.1 μm^−1^ for E1 and E2, respectively, resulting in a lower LoD, 0.40 and 0.30 ppb for E1 and E2, respectively (Figure 4). This connection between the nanocluster's S/V ratio and sensor sensitivity is an important finding for sensors, as shown in Figure 4. An AFM image of the patterned nanoclusters on electrodes E1 and E2 is shown in Figure 5a,b, whereas corresponding MATLAB‐processed images have been shown in Figure 5c,d. The elongated patterning arose due to the force applied during dwell time, causing the cantilever tip to slide a tiny distance along the *y*‐axis (Figure 5a,b). When a greater force was exerted on the electrode surface, the distance of the shift along the *y*‐axis increased. This can form an elliptical shape to the cluster base for some inks, which depends mainly on the cohesive–adhesive forces between the ink and the surface and the ink viscosity.

Besides the physical feature (S/V), which causes the difference in sensitivity, the nature of both ligands also impacts the sensitivity. Therefore, density functional theory (DFT) calculations were conducted to shed light on the strength of bonds between the lead as well as the other ligands with ink components, to explain the variation in sensitivity of each electrode (reaction 4–14). The calculations were performed according to the p*K*_a_ and the experimental conditions, which equals pH 3.^[^ ^46^, ^47^ ^]^ The p*K*_a_s of DAN are −0.1 and 4.0 for pK_a1_ and pK_a2_, respectively.^[^ ^46^ ^]^ Therefore, most species in the nanoclusters are DANH^+^ (DAN marked as DANH^+^ in acidic conditions) and only small amounts of DAN. The p*K*_a_s of D‐PA are 1.8, 7.9, and 10.5 for p*K*_a1_, p*K*_a2_, and p*K*_a3_, respectively;^[^ ^48^ ^]^ therefore, most species in the solution are D‐PA^−^. The structures of the obtained complexes are listed in **Table** **2** and S1, Supporting Information. The monomer (i.e., methyl methacrylate) was used as a polymer model; it was marked as PMMA; a better model of the polymer was a structure of three molecules of the monomer, marked TRIPMMA. The endergonic value of Δ*G* ^0^ indicated that the DAN does not react with PMMA, reaction S1, Supporting Information. However, in acidic conditions, the reaction between DANH^+^ and PMMA is isoenergetic (reaction S2, Supporting Information). The small Δ*G* ^0^ value of reaction S2, Supporting Information indicates that some species of PMMADANH^+^ are present. It is important to note that the tendency of the calculated Δ*G* ^0^ values with the monomer (PMMA) and the trimer (TRIPMMA), e.g., is similar, reaction 4, and S2, Supporting Information and for PMMA and TRIPMMA, respectively.

(4) $$\text{TRIPMMA}+{\text{DANH}}^{+}\to \text{DANH}\_{\text{TRIPMMA}}^{+}\;\Delta G^{0}=0.28\text{ kcal}\;\;{\text{mol}}^{-1}\;$$

(5) $$\text{Pb}{\left(H_{2}O\right)}_{3}^{2+}+\text{ TRIPMMA }\to {\left[\text{Pb}{\left(H_{2}O\right)}_{3}\_\text{TRIPMMA}\right]}^{2+}\Delta G^{0}=9.36\text{ kcal}\;\;{\text{mol}}^{-1}$$

(6) $$\text{Pb}{\left(H_{2}O\right)}_{3}^{2+}\;+{\text{ DANH}}^{+}\to \text{Pb}{\left(H_{2}O\right)}_{3}{\text{DANH}}^{3+}\left(C\right)\;\Delta G^{0}=5.98\text{ kcal}\;\;{\text{mol}}^{-1}\;$$

(7) $$\text{Pb}{\left(H_{2}O\right)}_{3}^{2+}\;+{\text{ DANH}}^{+}\;\to \text{Pb}{\left(H_{2}O\right)}_{3}{\text{DANH}}^{3+}\left(N\right)\;\Delta G^{0}=11.31\text{ kcal}\;\;{\text{mol }}^{-1}\;$$

**Table 2:** Structures used and calculated during the DFT study.

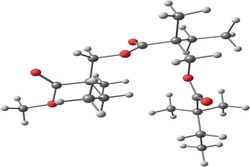	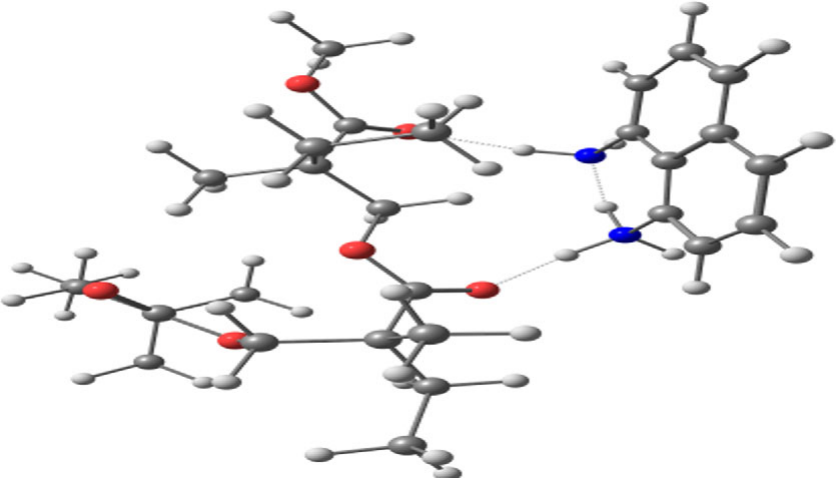
TRIPMMA (reaction 4)	DANH_TRIPMMA^+^ (reaction 4)
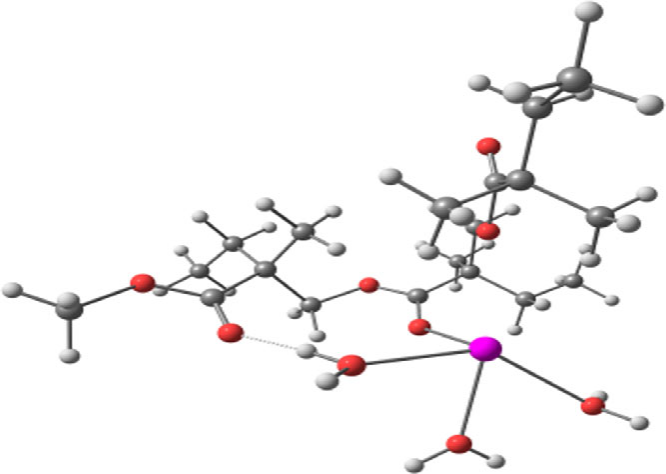	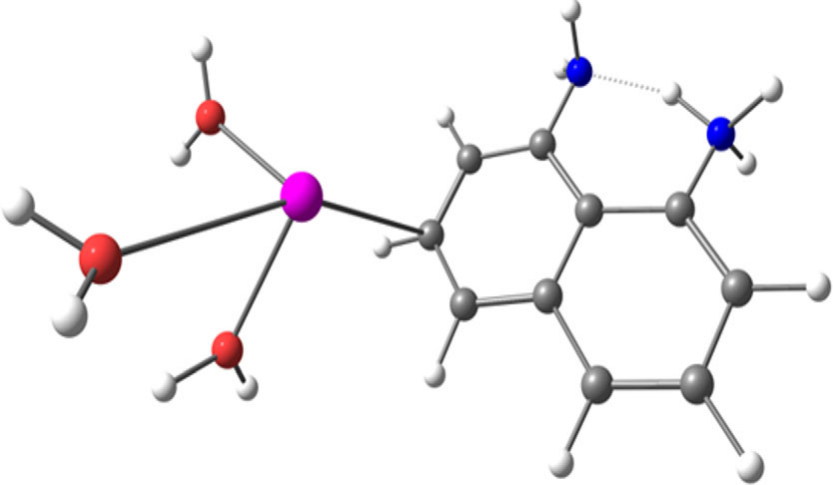
[Pb(H_2_O)_3__TRIPMMA]^2+^ (reaction 5)	Pb(H_2_O)_3_DANH^3+^ (C) (reaction 6)
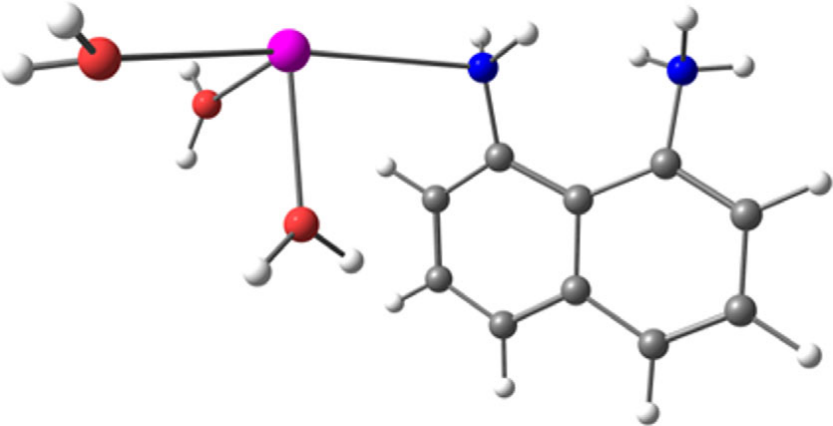	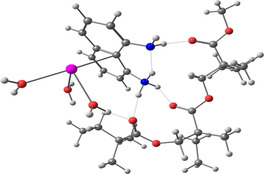
Pb(H_2_O)_3_DANH^3+^ (N) (reaction 7)	[Pb(H_2_O)_3_DANH(C)_TRIPMMA]^3+^ (reaction 8)
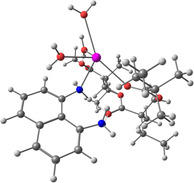	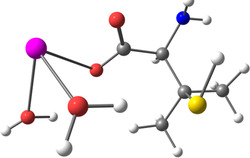
[Pb(H_2_O)_3_DANH(N)_TRIPMMA]^3+^ (reaction 9)	Pb(H_2_O)_2_D_PA^+^ (reaction 10)
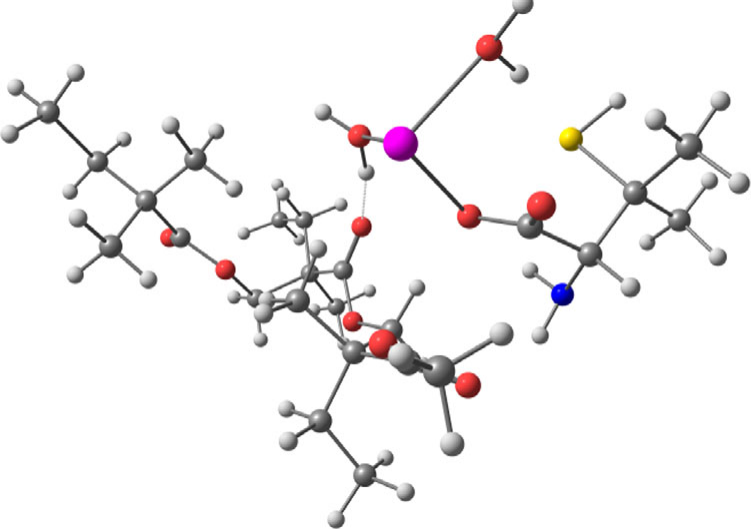	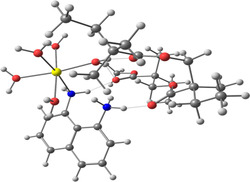
[Pb(H_2_O)_2_D_PA_TRIPMMA]^+^ (reaction 11)	[Cd(H_2_O)_5_DANH(N)_TRIPMMA]^3+^ (reaction 12)
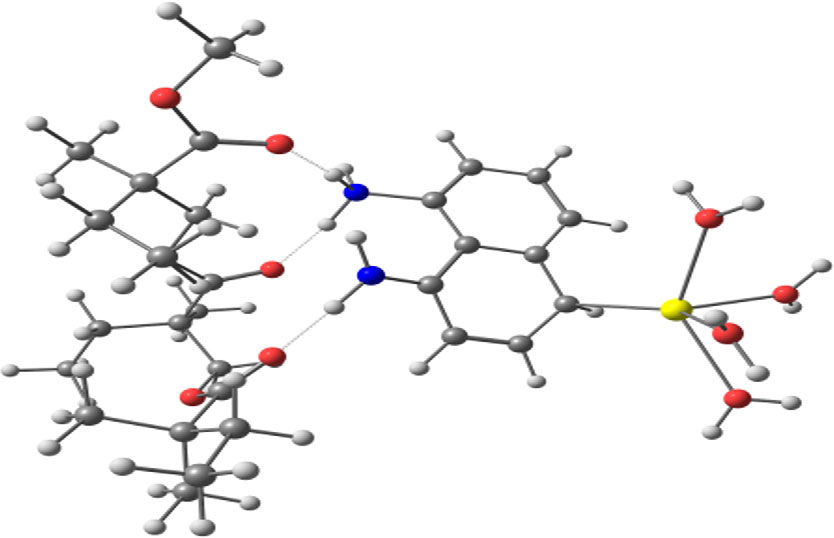	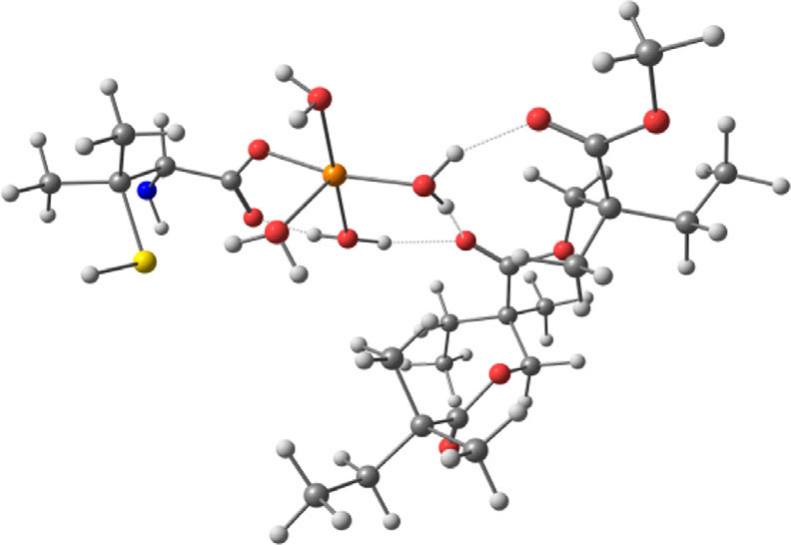
[Cd(H_2_O)_4__DANH(C)_TRIPMMA]^3+^ (reaction 13)	[Cu(H_2_O)_4_D_PA_TRIPMMA]^+^ (reaction 14)
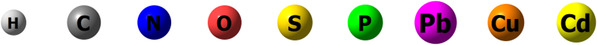	

The DFT study indicated that the lead did not bind to PMMA (as published earlier^[^ ^22^ ^]^) as well as to TRIPMMA (reaction 5, S3, Supporting Information, respectively). Also, the lead did not bind to the DANH^+^ ligand irrespective of the carbon or nitrogen site (reaction 6–7). The binding of lead to DANH^+^ via the carbon is less endergonic than the nitrogen because; as the lead binds to the nitrogen, it opens a hydrogen bond, which stabilizes the structure. According to the SWASV result (Figure 1a), there is an electrochemistry signal in the presence of DANH^+^; without it, there is no bounding with the ligands, so no electrochemical signal appears (reaction 5–7). There are several reports on the binding of lead to poly(DAN) in acidic conditions;^[^ ^41^, ^49^ ^]^ however, reports have yet to be available on the binding between DAN and lead. Therefore, and according to the endergonic results obtained, DFT calculations for the binding of di‐DAN and diDANH^+^ (model for the polyDAN) with lead were performed (reaction S4–S5, Supporting Information), and it was found that this reaction is possible. However, in our experimental conditions, there was no polymerization reaction of DAN.^[^ ^50^ ^]^ The possibility of binding between lead and PMMADAN, PMMADANH^+^ (reaction S6–S7, Supporting Information), and TRIPMMA with DANH^+^ was also investigated (reaction 8–9). The calculations indicated that the binding of the cations to PMMADAN exists and that they are stronger than the binding to PMMADANH^+^, reaction S6–S7, Supporting Information, respectively. In the case of PMMADANH^+^, the reaction is between two positively charged species that repel each other; therefore, their binding is endergonic. However, the Δ*G* ^0^ value of reaction S7, Supporting Information, is small; this reaction is almost isoenergetic; thus, it indicates that a small portion of the lead binds to the PMMADANH^+^ (the existing species in the solution according to the experimental conditions, pH 3). The tendency reverses when there is TRIPMMA (reaction 8–9) compared to reaction 6–7. In the presence of TRIPMMA, an intermolecular hydrogen bond is formed between the hydrogen of the amines and the TRIPMMA's oxygens, compensating for the intramolecular hydrogen bonding disconnection between the two amine groups in the DANH^+^ when the lead binds to the nitrogen (the same is true for Cd^2+^, reaction 12–13). As Δ*G* ^0^ of reaction 9 is very small, the reaction is almost isoenergetic, indicating that some lead binds to DANH^+^ in the presence of TRIPMMA.

(8) $$\text{Pb}{\left(H_{2}O\right)}_{3}^{2+}+\text{ TRIPMMA}+{\text{DANH}}^{+}\to {\left[\text{Pb}{\left(H_{2}O\right)}_{3}\_\text{DANH}\left(C\right)\_\text{TRIPMMA}\right]}^{3+}\;\;\;\;\;\;\;\;\;\;\;\Delta G^{0}=9.21\text{ kcal}\;\;{\text{mol}}^{-1}$$

(9) $$\text{Pb}{\left(H_{2}O\right)}_{3}^{2+}+\text{ TRIPMMA}+{\text{DANH}}^{+}\to {\left[\text{Pb}{\left(H_{2}O\right)}_{3}\_\text{DANH}\left(N\right)\_\text{TRIPMMA}\right]}^{3+}\;\Delta G^{0}=2.21\text{ kcal}\;\;{\text{mol }}^{-1}$$

**Figure 6:**
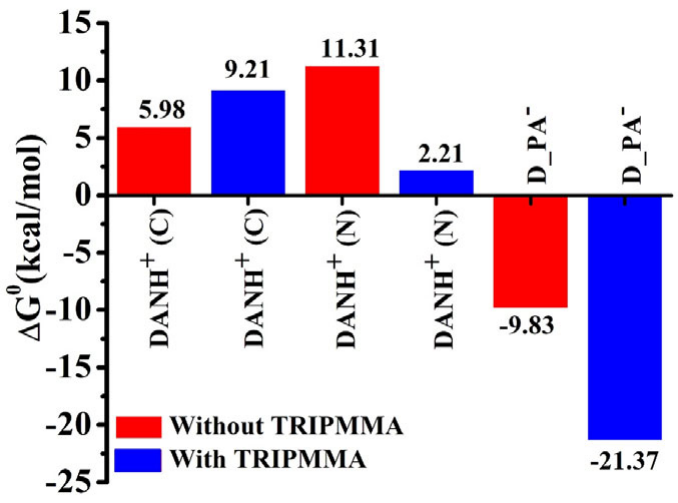
Gibbs’ free energy (Δ*G* ^0^) for Pb(II) binding with DANH^+^ and D‐PA ligands in the presence (blue columns) and absence (red columns) of TRIPMMA.

Due to the large S/V ratio and the presence of the PMMA, which increases the binding strength (**Figure** **6**), the lead binds to the DANH^+^, and a redox peak was observed, and the sensor could detect low lead concentrations. Also, the calculations were done without considering the voltage applied, which happened in practice during the sample detection and helped the reaction to occur. Therefore, we detected oxidation peaks during sensing. Additionally, the sensing performance was investigated also using D‐PA. D‐PA has three functional groups that could bind lead: carbonyl through O, sulfide via S, and amine via N. The DFT calculation showed that each functional group binds lead (the carboxyl, sulfide and amine groups), reaction S8 and S9, Supporting Information; however, the binding is highly favorable for the carboxyl group, which is deprotonated (p*K*_a_ of the carboxylic group is 1.8, at our experimental conditions pH 3, Δ*G* ^0^ = −9.83 kcal mol^−1^; (reaction 10),^[^ ^48^ ^]^ compared to the sulfide and amine groups, reaction S8 and S9, Supporting Information, respectively. The DFT study shows that PMMA with D‐PA played a substantial and interesting role in binding the lead besides its role in the patterning process.^[^ ^51^ ^]^ The binding reaction of lead in the presence of PMMA and TRIPMMA is more exergonic (reaction 10, 11 and S11, Supporting Information).

(10) $$\text{Pb}{\left(H_{2}O\right)}_{3}^{2+}\;+{\text{ D}}_{-}{\text{PA}}^{-}\;\to \text{Pb}{\left(H_{2}O\right)}_{2}D_{-}{\text{PA}}^{+}\left(O\right)+{\text{ H}}_{2}\text{O }\Delta G^{0}=-9.83\text{ kcal}\;\;{\text{mol }}^{-1}$$

(11) $$\text{Pb}{\left(H_{2}O\right)}_{3}^{2+}+\text{TRIPMMA}+D_{-}{\text{PA}}^{-}\;\to {\left[\text{Pb}{\left(H_{2}O\right)}_{2}\_D_{-}{\text{PA}}_{-}\text{TRIPMMA}\right]}^{+}\;+{\text{ H}}_{2}\text{O }\Delta G^{0}=-21.37\text{ kcal}\;\;{\text{mol}}^{-1}$$

Also, this finding motivated us to calculate the binding of the cations with NTPH, which had been used in our previous work,^[^ ^22^ ^]^ and we found that PMMA enhanced the binding of lead in the presence of NTPH, as more negative free energy was obtained, reaction S11^[^ ^22^ ^]^ and S12, Supporting Information. This result indicated that the PMMA, which we thought was inert and whose role is only to be used in the patterning process as a carrier for the ligand and a “glue” used to bind the nanoclusters to the electrode's surface, must also be considered a functional part of the detection mechanism.^[^ ^22^ ^]^ It has a vital role in lead binding and, as a result, an essential role in the detection process. We assume that the charge dispersion via hydrogen bonds between the ligands and the oxygen of the PMMA^[^ ^52^ ^]^ makes the binding between lead and the ligands more favorable for sensing, which might amplify the signal. The Δ*G* ^0^ of all the ligands with lead is included in Figure 6.

**Figure 7:**
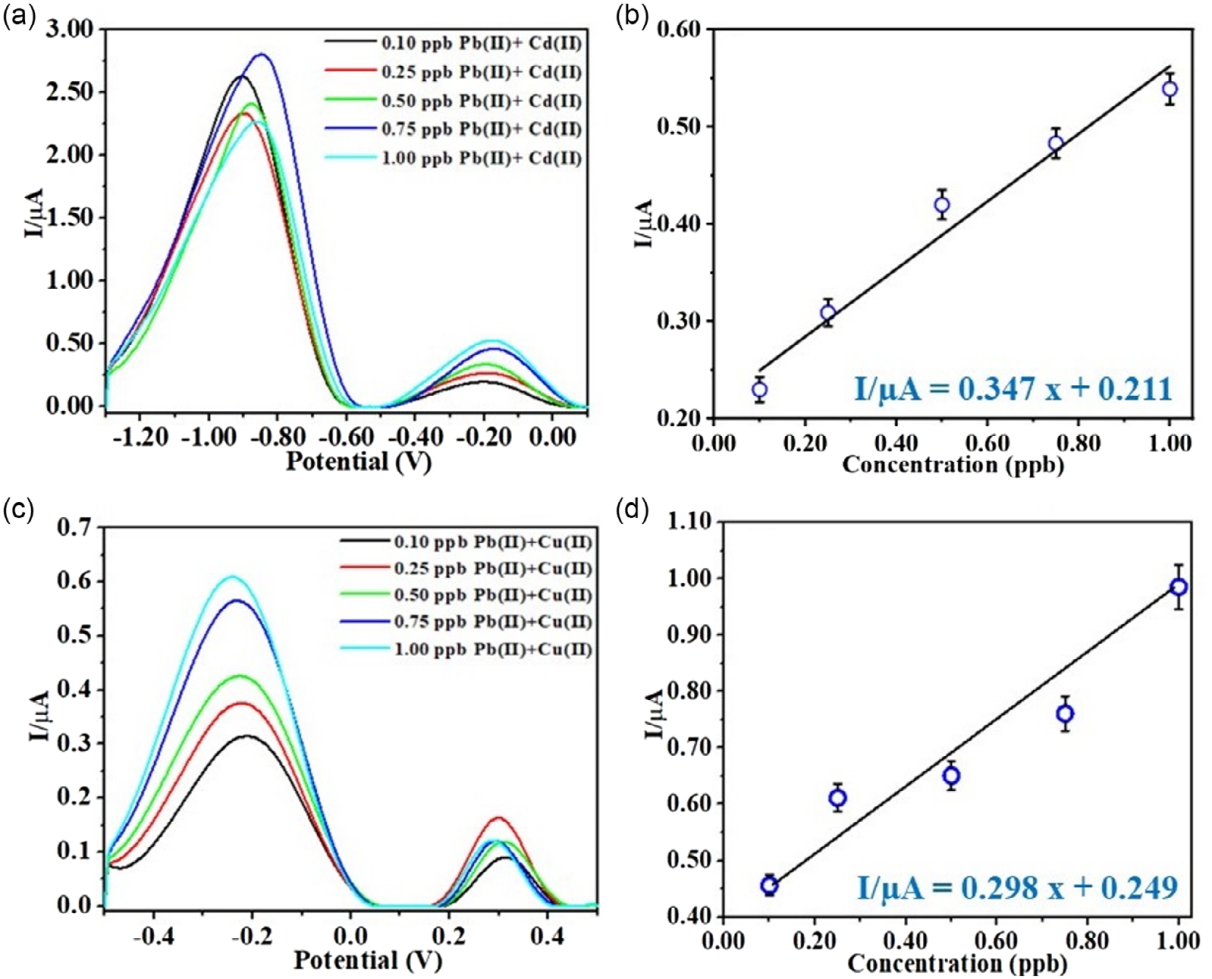
Multication detection via a) SWASV of Pb(II) in the presence of 0.10 ppb Cd(II) for E1, b) calibration of Pb(II) in the presence of 0.10 ppb Cd(II) for E1, c) SWASV of Pb(II) in the presence of 0.10 ppb Cu(II) for E2, and d) calibration of Pb(II) in the presence of 0.10 ppb Cu(II) for E2. All experiments were performed at pH 3.0 and 100 μM ionic strength (E deposition: −1.1 V, deposition time: 60 s, step potential: 5 mV, Amplitude: 100 mV, Frequency deposition: 25 Hz).

We tested our electrodes (sensors), E1 and E2, to detect Pb(II) in the presence of other cations. E1 was used for the detection of Pb(II) in the presence of 0.10 ppb Cd(II), whereas E2 was used for Pb(II) detection in the presence of 0.10 ppb Cu(II) (**Figure** **7**). The SWASV showed that the oxidation peak for Pb(II) increased continuously with the increased concentration of Pb(II). In contrast, the Cd(II) and Cu(II) oxidation peaks are almost stable (Figure 7a,c). The calibration for Pb(II) in the presence of Cd(II) and Cu(II) was plotted and fitted with linear equations: *I*/μA = 0.347*x* + 0.211 (*R* ^2^ = 0.97) and *I*/μA = 0.298*x* + 0.249 (*R* ^2^ = 0.96), respectively (Figure 7b,d). The LoD values were calculated from the calibration plot, and they were found to be 0.43 and 0.42 ppb for Pb(II) in the presence of Cd(II) and Cu(II), respectively. The obtained LoD increased relatively compared to single lead sensing in the solution, as expected when more than one metal exists (**Table** **3**).^[^ ^53^ ^]^

**Table 3:** LoD for Pb(II) in the presence and absence of other cations.

Electrode name	LoD of Pb(II) (single‐element solution)	LoD of Pb(II) (Multielement solution)
E1 (DAN‐based electrode)	0.40	0.43 [with 0.10 ppb of Cd (II)]
E2 (D‐PA‐based electrode)	0.30	0.42 [with 0.10 ppb of Cu (II)]

The binding of Cd(II) and Cu(II) to the DANH^+^ and D‐PA could explain the sensitivity tendency of the sensor for Pb(II) (reaction 12–14). When the solution includes Cd(II) and Pb(II), the LoD stays almost similar compared to a solution only with Pb(II). Because Pb(II) binds to DANH^+^ better than Cd(II) (reaction 6–9, 12–13, and S13–S16, Supporting Information), there is no competition between those cations. However, the sensitivity of the sensor for Pb(II) decreases in the presence of Cu(II) in the solution because Pb(II) binds to the D‐PA and Cu(II) binds better (reaction 10, 11, 14, and S17, Supporting Information). Therefore, competition between those cations causes a decrease in the sensitivity of Pb(II), Table 3.

(12) $$\text{Cd}{\left(H_{2}O\right)}_{6}^{2+}+\text{TRIPMMA}+{\text{DANH}}^{+}\to {\left[\text{Cd}{\left(H_{2}O\right)}_{5}\text{DANH}{\left(N\right)}_{-}\text{TRIPMMA}\right]}^{3+}+H_{2}\text{O }\Delta G^{0}=2.81\text{ kcal}\;{\text{mol }}^{-1}\;$$

(13) $$\text{Cd}{\left(H_{2}O\right)}_{6}^{2+}+\text{TRIPMMA}+{\text{DANH}}^{+}\to {\left[\text{Cd}{\left(H_{2}O\right)}_{4}\text{DANH}{\left(C\right)}_{-}\text{TRIPMMA}\right]}^{3+}+2H_{2}\text{O }\Delta G^{0}=19.59\text{ kcal}\;\;{\text{mol}}^{-1}\;$$

(14) $$\text{Cu}{\left(H_{2}O\right)}_{6}^{2+}+\text{TRIPMMA}+D_{-}{\text{PA}}^{-}\to {\left[\text{Cu}{\left(H_{2}O\right)}_{4}D_{-}{\text{PA}}_{-}\text{TRIPMMA}\right]}^{+}+2H_{2}\text{O }\Delta G^{0}=-29.31\text{ kcal}\;\;{\text{mol }}^{-1}$$

The LoD values obtained in this current study are in close agreement with those published in the earlier literature (Table S2, Supporting Information). The lowest reported LoD for Pb(II) is 0.11 μM for a Bi‐based working electrode.^[^ ^54^ ^]^ The reported electrodes for Pb(II) sensing were modified with either drop‐casting or material deposition over working electrodes,^[^ ^3^, ^45^ ^]^ whereas our work presents a novel DPN patterning of electrodes.

## 3 Conclusions

The discussed research focused on detecting trace amounts of multiple cations, including Pb(II), at a pH of 3.0. One of this study's innovations is the DPN technique, which was used to pattern the DAN‐ and D‐PA‐based inks over gold surfaces. The high control over patterning resulted in highly efficient MCS electrodes with higher sensitivity compared to other heavy metal sensors. It was seen that the D‐PA‐based electrode (E2) had a lower LoD than the DAN‐based electrode (E1) due to stronger D‐PA binding of the ligand and the larger S/V ratio of the D‐PA nanoclusters compared to DAN nanoclusters. Further, multication trace elements were detected using MCS sensors, showing that the DAN‐based MCS is effective for detecting Pb(II) in the presence of Cd(II) and the D‐PA‐based MCS is good for Pb(II) in the presence of Cu(II). The lead sensitivity decreased in the presence of copper in the solution because of the competition between the metals. That was quantified for the first time in a sensor system by Δ*G* ^0^ values obtained with DFT calculations. Another important, intriguing, and applicable result regarding the PMMA role was found via DFT calculations: PMMA increased the heavy metals’ binding strength to the ligands; without it, the lead did not bind to the DANH^+^. This work is the first to report and address this unique and exciting property of the PMMA role, which could be applied to increasing detection sensitivity and controlling heavy metal detection.

## 4 Experimental Section

4.1

4.1.1

##### Materials

All the materials used for patterning and electrochemical analysis were purchased from Sigma–Aldrich in AR grade and were used as received: DAN (C_10_H_6_(NH_2_)_2_), D‐PA (C_6_H_11_NO_3_S), Pb(NO_3_)_2_, Cu(NO_3_)_2_, Cd(NO_3_)_2_, ACN, sodium acetate buffer (pH 4.5), nitric acid (69%), and PMMA (Northland Optical Adhesive 68T, USA). For the patterning, a thin film of gold electrode (SE1 (AuAuAu)) was purchased from MicruX Technologies, Spain (Figure S2, Supporting Information). Ultrapure water purified by a Treka‐type TKA‐GenPure system with a final resistance of 18.2 MΩ cm was used.

##### Instruments

For patterning the MCSs, the DPN platform NLP 2000 (NanoInk Inc., IL) was utilized, enabling precision in AFM resolution. The clusters were created using a 1D tip array (12 tips with a 66 μm pitch between tips; M‐type, NanoInk). The tip shape affects the shape of the obtained clusters, and any dust on tips can alter the patterned clusters; therefore, before the pattering process, the 1D tip array was cleaned in oxygen plasma using a plasma cleaner (Plasma Preen 2 medium chamber, USA). The patterned nanoclusters were cured with 365 nm wavelength UV light for ≈20 min to finalize cross‐linking. The surface of the patterned electrode was scanned by an AFM (Easy Scan 2 Flex AFM, NanoSurf, Liestal, Switzerland) in tapping mode. Scanning electron microscopy (SEM, FEI, Thermo Fisher Scientific, variable 400 L, Hillsboro, OR, USA) was utilized for the surface morphology of patterned clusters. The electrochemistry study was performed over a miniature USB‐powered potentiostat (EmStat3, PalmSens) with PSTrace 5.9 data analyzer software.

##### Ink Preparation

Three types of ink were prepared based on ligand and ink constituents; the ink compositions are summed up in **Table** **4**. Briefly, 500 μL PMMA (a commercial mixture of 70–95% mercapto esters and 5–30% tetrahydrofurfuryl methacrylate) was added into 500 μL ACN, followed by ultrasonication for 20 min. Then, DAN or D‐PA was added, followed by another 10 min of sonication. PMMA was chosen because it is known as a very suitable polymer for patterning by NLP2000; its viscosity allows it to flow well during the patterning process.^[^ ^22^ ^]^ The prepared inks were kept in dark and cold storage due to the polymerization of PMMA in light^[^ ^22^ ^]^ and the evaporation of ACN in warmth. Before using the ink to fabricate the MCS electrodes, the prepared ink was ultrasonicated for 5 min, followed by a vortex mixer.

**Table 4:** Ink's composition table.

Name of ink	Ink composition			
	PMMA [μL]	Acetonitrile [μL]	Ligand	
			DAN [mg]	D‐PA [mg]
Ink‐1	500	500	16	–
Ink‐2			–	16
Ink‐3	500	500	–	–

##### Patterning Procedures (for E1, E2, and E3 Electrodes)

The MSC electrode was fabricated with an NLP2000 nanolithography platform (NanoInk, Inc.) using a 1D tip array with 12 tips spaced 66 μm apart (M‐type). The prepared ink was placed into the inkwells, and the tips were loaded with ink by bringing the tips near the inkwell microchannels with a controlled scanning probe setup. The loaded tips with the selected ink were placed in the desired location on the patterned surface, and ink was deposited onto the substrate through a meniscus. The tips were adjusted for uniform patterning so that all the tips touched the substrate simultaneously. It is also important that uniform amounts of ink are loaded; therefore, the tips were aligned to touch the inkwell capillaries simultaneously with the same force. During the patterning, the tips touched the substrate (dwell time) for 1 s. The temperature and relative humidity also play a significant role; these were fixed at ≈18–19 °C and 40–42%, respectively. After completion, the patterned MCS's were cured under a 365 nm UV lamp for 20 min for the final cross‐linking of PMMA (ink component). Table 1 summarizes the various electrodes. Some surfaces were scanned by SEM, besides the AFM scanning, before and after the electrochemical experiments (Figure S3a,b, Supporting Information). The SEM scan before the electrochemical study showed uniform and fine patterning; however, after sensing performance, nanoparticles from electrolytes were deposited over the surface, making patterns a little darker.

##### Coating Procedures by NLP2000 (for E1‐1 and E2‐1 Electrodes Preparation)

Uniform coating was done by NLP2000 with two types of inks, ink‐1 and ink‐2 using an M‐type probe. The NLP 2000 produced a uniform layer with a thickness approximately equal to the average nanocluster's height, 220 nm, and 132 nm height, with ink‐1 and ink‐2, respectively. The temperature and relative humidity were fixed at ≈18–19 °C and 40–42%, respectively. After the coating process, the uniformly coated electrodes were cured under a 365 nm UV lamp for 20 min for the final cross‐linking of PMMA.

##### DFT Calculations

DFT calculations at the level of B3LYP/6‐311 + G(d,p) (SDD^[^ ^55^ ^]^ for transition metals^[^ ^56^ ^]^) were carried out using the Gaussian16 program.^[^ ^57^ ^]^ Grimme's D3BJ dispersion with Becke–Johnson damping^[^ ^58^ ^]^ was considered in all calculations. Vibrational frequencies were calculated for each structure to confirm all stationary points’ nature and obtain the associated thermodynamic energy corrections. The solvation effects were included by using the SMD^[^ ^59^ ^]^ implicit solvation model, and standard‐state concentration corrections^[^ ^60^ ^]^ were added to the computed free energies. As in the previous case,^[^ ^22^ ^]^ in the DFT calculation, the Pb^2+^ is three coordinated (Pb(H_2_O)_3_^2+^).^[^ ^61^, ^62^, ^63^, ^64^, ^65^ ^]^ Chemcraft software was used to capture the 3D image.^[^ ^66^ ^]^

## Conflict of Interest

The authors declare no conflict of interest.

## Author Contributions

**Rahma Okbi**: Data curation (equal). **Mohammed Alkrenawi**: Data curation (equal). **Krishna Kumar Yadav**: Writing—original draft (equal). **Dror Shamir**: Data curation (lead); Investigation (equal); Methodology (equal); Supervision (lead). **Haya Kornweitz**: Data curation (lead). **Yael Peled**: Visualization (equal). **Moshe Zohar**: Data curation (lead); Investigation (equal); Methodology (equal); Software (lead). **Ariela Burg**: Investigation (equal); Methodology (lead); Project administration (lead); Supervision (lead); Writing—original draft (lead); Writing—review and editing (lead).

## Supporting information

Supplementary Material

## Data Availability

The data that support the findings of this study are available in the supplementary material of this article.
