# Supplementary material for: Dip‐Pen Nanolithography‐Based Fabrication of Meta‐Chemical Surface for Heavy Metal Detection: Role of Poly‐Methyl Methacrylate in Sensor Sensitivity
Source: Small Sci. 2024 Nov 20;5(2):2400459. doi: 10.1002/smsc.202400459 (PMC11934891; doi:10.1002/smsc.202400459)
Supplement: Supplementary file 1 — Supplementary Material [file SMSC-5-2400459-s001.pdf]

Supporting Information

Dip-Pen Nanolithography-based Fabrication of Meta Chemical Surface for Heavy Metal Detection: Role of PMMA in Sensor Sensitivity

Rahma Okbi, Mohammed Alkrenawi, Krishna K. Yadav, Dror Shamir, Haya Kornweitz, Yael Peled, Moshe Zohar and Ariela Burg[*]

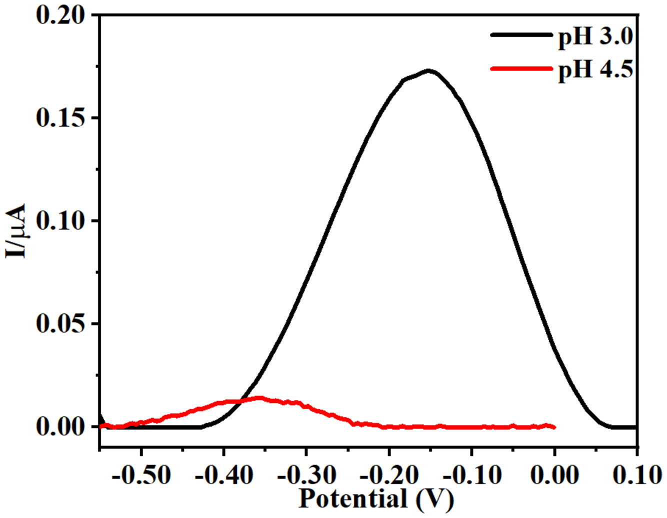

Figure S1. SWASV voltammograms of electrode E-1 for 0.25 ppb Pb(II), in different supporting electrolytes and pH: (black line) 100 µM HNO_3 at pH 3.0 and (red line) 100 µM acetate buffer at pH 4.5. Accumulation for 60 s at -1.1V, followed by a SWASV scanning with a step potential of 5 V, an amplitude of 100 mV, and a frequency of 25 Hz.

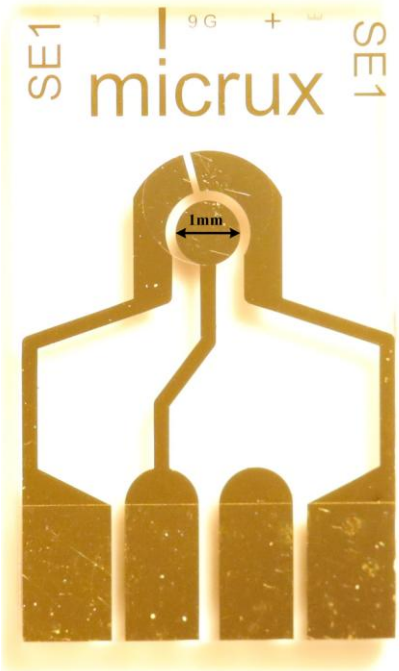

Figure S2. Gold MicruX electrode; the patterning was performed in the circled area (1mm diameter).

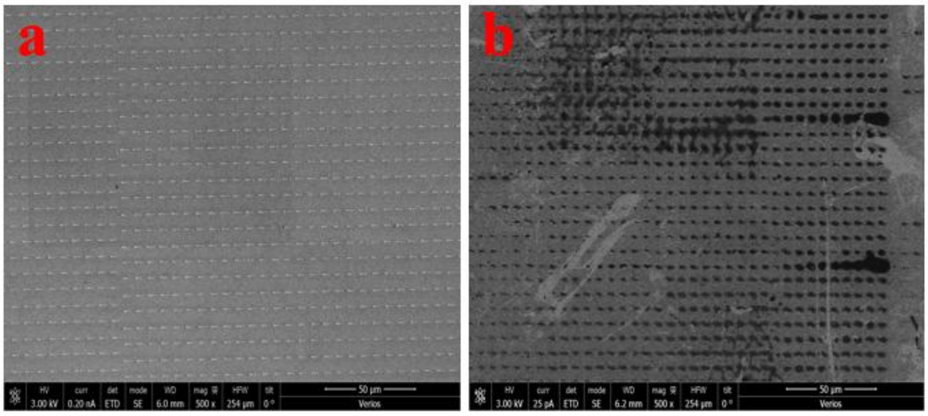

Figure S3. SEM micrograph of E1, (a) As patterned, (b) After electrochemical study.

List of reactions and ΔG^0 values that were obtained with DFT calculation.

$\mathrm{PMMA + DAN \ \rightarrow PMMADAN}$

$\Delta G^0 = 4.28\ \mathrm{kcal/mol}$ (S1)

$\mathrm{PMMA + DANH^+ \ \rightarrow PMMADANH^+}$

$\Delta G^0 = 0.70\ \mathrm{kcal/mol}$ (S2)

$\mathrm{Pb(H_2O)_3^{2+} \ + \ PMMA \ \rightarrow Pb(H_2O)_3PMMA^{2+}}$

$\Delta G^0 = 3.30\ \mathrm{kcal/mol}$ (S3)

$\mathrm{Pb(H_2O)_3^{2+} \ + \ diDAN \ \rightarrow Pb(H_2O)_3diDANH^{2+}}$

$\Delta G^0 = -0.86\ \mathrm{kcal/mol}$ (S4)

$Pb(H_2O)_3^{2+}\ +\ diDANH^{+}\ \rightarrow Pb(H_2O)_3diDANH^{3+}$

$\Delta G^0 = -2.16\ kcal/mol$ (S5)

$Pb(H_2O)_3^{2+}\ +\ PMMADAN\ \rightarrow Pb(H_2O)_3DANPMMA^{2+}$

$\Delta G^0 = -9.82\ kcal/mol$ (S6)

$Pb(H_2O)_3^{2+}\ +\ PMMADANH^{+}\ \rightarrow Pb(H_2O)_3DANHPMMA^{3+}$

$\Delta G^0 = 1.9\ kcal/mol$ (S7)

$Pb(H_2O)_3^{2+}\ +\ D\_PA(S)^{+}\ \rightarrow Pb(H_2O)_3D\_PA^{+}(S\ and\ O)$

$\Delta G^0 = -8.69\ kcal/mol$ (S8)

$Pb(H_2O)_3^{2+}\ +\ D\_PA(N)^{-}\ \rightarrow Pb(H_2O)_3D\_PA^{+}(N)$

$\Delta G^0 = -1.39\ kcal/mol$ (S9)

$Pb(H_2O)_3^{2+} + PMMAD\_PA^{-} \rightarrow Pb(H_2O)_2PMMAD\_PA^{+} + H_2O$

$\Delta G^0 = -17.61\ kcal/mol$ (S10)

$Pb(H_2O)_3^{2+} + NTPH^{3-} \rightarrow Pb(H_2O)_2NTPH^{-} +\ H_2O$

$\Delta G^0\ =\ -10.3\ kcal/mol$ [22] (S11)

$Pb(H_2O)_3^{2+} + PMMANTPH^{3-} \rightarrow Pb(H_2O)_3PMMANTPH^{-}$

$\Delta G^0\ =\ -24.76\ kcal/mol$ (S12)

$Cd(H_2O)_6^{2+}\ + DANH^{+}\ \rightarrow [Cd(H_2O)_4DANH(C)]^{3+} + 2H_2O$

$\Delta G^0 = 6.55\ kcal/mol$ (S13)

$Cd(H_2O)_6^{2+}\ + DANH^{+}\ \rightarrow [Cd(H_2O)_5DANH(N)]^{3+} + H_2O$

$\Delta G^0 = 7.57\ kcal/mol$ (S14)

$Cd(H_2O)_6^{2+}\ + PMMADANH^{+}\ \rightarrow Cd(H_2O)_5DANHPMMA^{3+} + H_2O$

$\Delta G^0 = 7.02\ kcal/mol$ (S15)

$Cd(H_2O)_6^{2+}\ + PMMADAN\ \rightarrow Cd(H_2O)_5DANPMMA^{2+} + H_2O$

$\Delta G^0 = -6.65\ kcal/mol$ (S16)

$Cu(H_2O)_6^{2+} + PMMAD\_PA \rightarrow Cu(H_2O)_2PMMAD\_PA + 4H_2O$

$\Delta G^0 = -39.88\ kcal/mol$ (S17)

Table S1. Structures used and calculated during the DFT study.

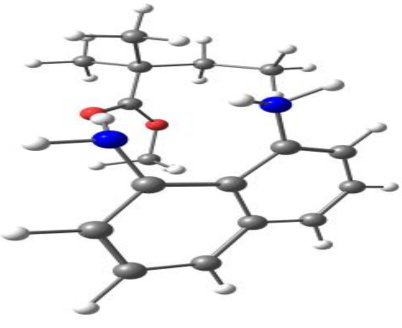	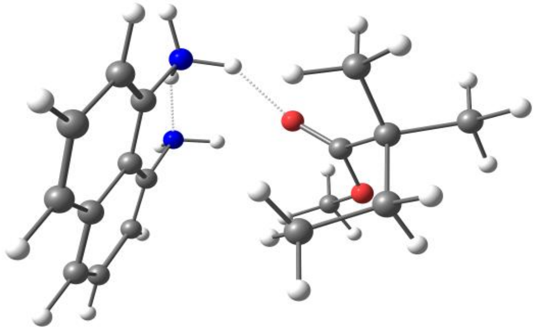
PMMADAN (reaction S1)	$PMMADANH^{+}$ (reaction S2)
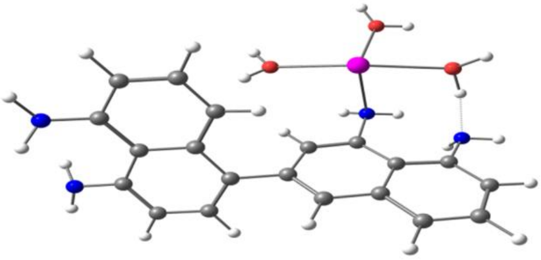	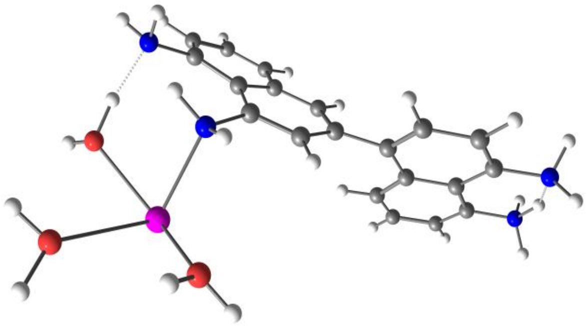
$Pb(H_2O)_3diDAN^{2+}$ (reaction S4)	$Pb(H_2O)_3diDAN^{3+}$ (reaction S5)
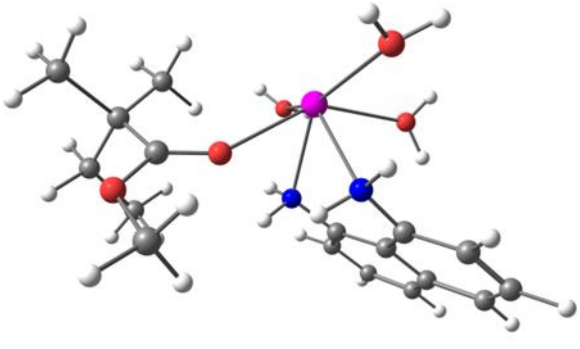	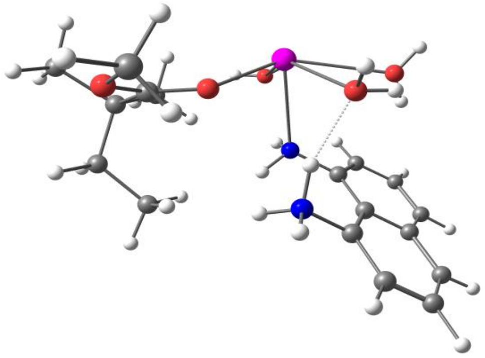
$Pb(H_2O)_3DANPMMA^{2+}$ (reaction S6)	$Pb(H_2O)_3DANHPMMA^{3+}$ (reaction S7)
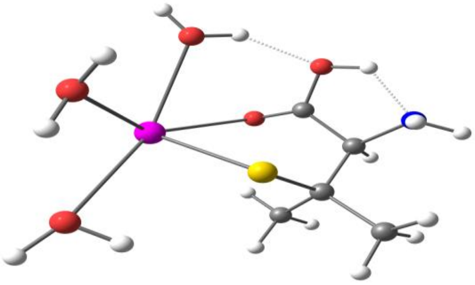	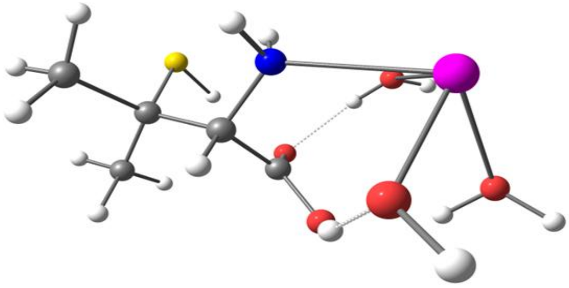
$Pb(H_2O)_3D\_PA^{+}$ (S, O) (reaction S8)	$Pb(H_2O)_3D\_PA^{+}$ (N) (reaction S9)

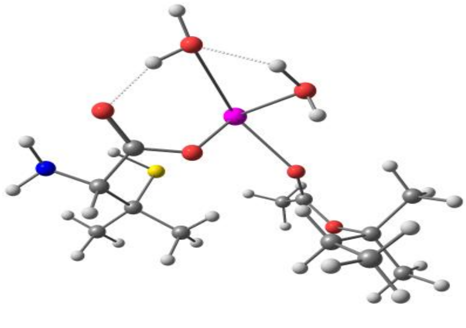

Pb(H_2O)$_2$PMMAD_PA^{+} (reaction S10)

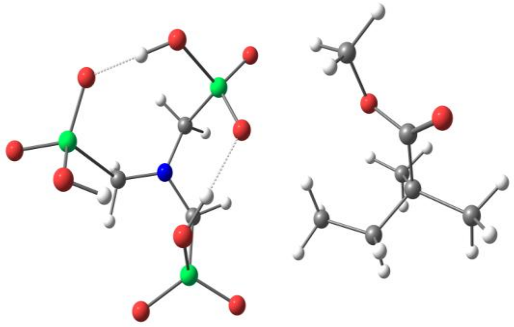

PMMANTPH^{3-} (reaction S12)

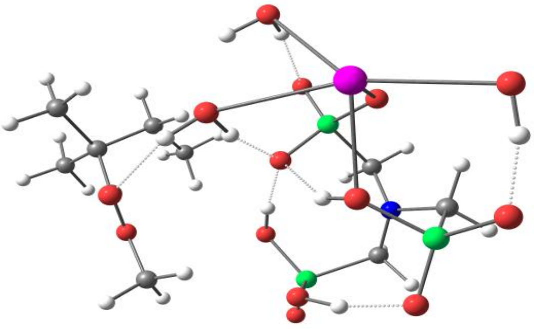

Pb(H_2O)$_3$ PMMANTPH^{-} (reaction S12)

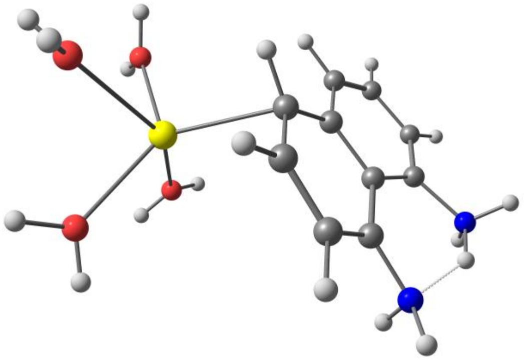

[Cd(H_2O)$_4$_DANH(C)]$^{3+}$ (reaction S13)

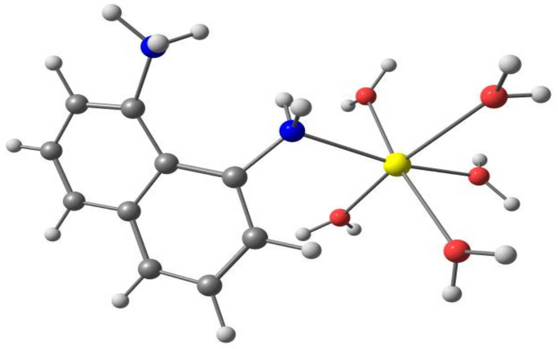

[Cd(H_2O)$_5$_DANH(N)]$^{3+}$ (reaction S14)

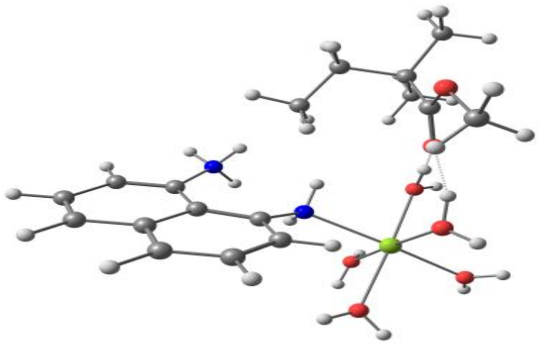

Cd(H_2O)$_5$DANHPMMA^{3+} (reaction S15)

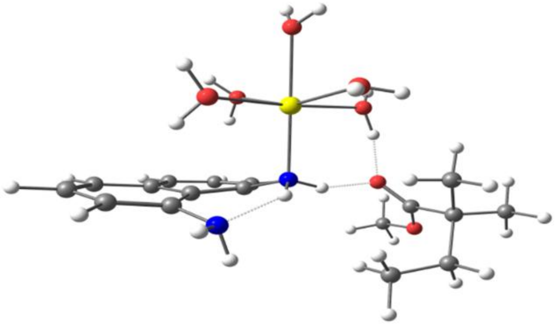

Cd(H_2O)$_5$DANPMMA^{2+} (reaction S16)

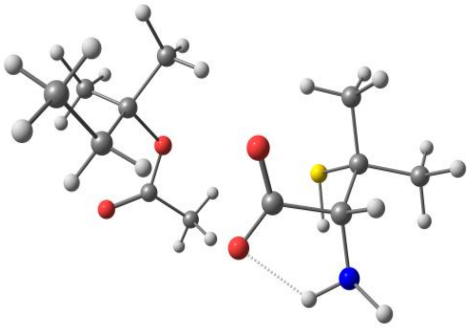

PMMAD_PA (reaction S17)

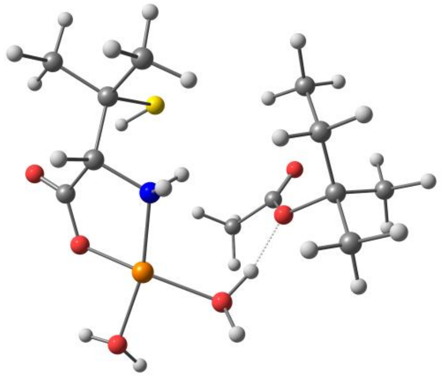

$Cu(H_2O)_2$PMMAD_PA (reaction S17)

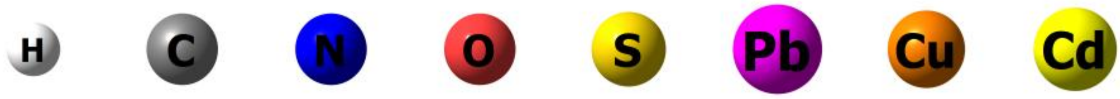

Table S2. Limit of detection (LoD) for various modified electrodes.

Modified Electrode	Methods	LoD (µg/L) Pb (II)	Reference
Bi film/crown ether/Nafion/SPCE	-	0.11 [in the presence of Cd(II)]	[2]
MWCNTs/synthesis Schiff base/CPE	SWASV	0.25 ng mL^{-1} [in the presence of Cd(II)]	[3]
Diacetyldioxime/CPE	differential pulse stripping voltammetry	2.07 [in the presence of Cd(II)]	[4]
TiO_2/ZrO_2 composite/CPE	SWASV	0.48 [in the presence of Cd(II)]	[5]
G/PANI/PS nanoporous fiber/SPCE	SWASV	3.30 [in the presence of Cd(II)]	[6]
Bi-CNT/SPCE	anodic stripping voltammetry	1.3 [in the presence of Cd(II)]	[7]
P(DPA-co-2ABN)/GC	Differential pulse voltammetry	165 [in the presence of Cd(II)]	[8]
AuNPs/PANI-MWCNTs/SPCE	SWASV	0.037 [in the presence of Cu(II)]	[9]
AuNPs/SPGE	SWASV	2.2 [in the presence of Cu(II)]	[10]
Bi/AuNP/SPCE	differential pulse anodic stripping voltammetry	0.027 [in the presence of Cu(II)]	[11]
Au@pani/GCE	SWASV	0.062 [in the presence of Cu(II)]	[12]

References

[1] K. K. Yadav, D. Shamir, H. Kornweitz, Y. Peled, M. Zohar, A. Burg, *Small Methods* **2023**, *8*, 2301118.

[2] K. Keawkim, S. Chuanuwatanakul, O. Chailapakul, S. Motomizu, *Food Control* **2013**, *31*, 14.

[3] A. Afkhami, H. Ghaedi, T. Madrakian, M. Rezaeivala, *Electrochim Acta* **2013**, *89*, 377.

[4] C. Hu, *Talanta* **2003**, *60*, 17.

[5] P. K. Q. Nguyen, S. K. Lunsford, *Journal of Electroanalytical Chemistry* **2013**, *711*, 45.

[6] N. Promphet, P. Rattanarat, R. Rangkupan, O. Chailapakul, N. Rodthongkum, *Sens Actuators B Chem* **2015**, *207*, 526.

[7] G. HWANG, W. HAN, J. PARK, S. KANG, *Talanta* **2008**, *76*, 301.

[8] M. F. Philips, A. I. Gopalan, K.-P. Lee, *J Hazard Mater* **2012**, *237–238*, 46.

[9] Y. Shao, Y. Dong, L. Bin, L. Fan, L. Wang, X. Yuan, D. Li, X. Liu, S. Zhao, *Microchemical Journal* **2021**, *170*, 106726.

[10] H. Wan, Q. Sun, H. Li, F. Sun, N. Hu, P. Wang, *Sens Actuators B Chem* **2015**, *209*, 336.

[11] Z. Lu, J. Zhang, W. Dai, X. Lin, J. Ye, J. Ye, *Microchimica Acta* **2017**, *184*, 4731.

[12] Z. Lu, W. Dai, B. Liu, G. Mo, J. Zhang, J. Ye, J. Ye, *J Colloid Interface Sci* **2018**, *525*, 86.
